# The use of theories, frameworks, or models in knowledge translation studies in healthcare settings in China: a scoping review protocol

**DOI:** 10.1186/s13643-020-01567-4

**Published:** 2021-01-07

**Authors:** Junqiang Zhao, Xuejing Li, Lijiao Yan, Yamei Yu, Jiale Hu, Shelly-Anne Li, Wenjun Chen

**Affiliations:** 1 School of Nursing, University of Ottawa, Ottawa, Ontario Canada; 2 Center for Research on Health and Nursing, University of Ottawa, Ottawa, Ontario Canada; 3 School of Nursing, Beijing University of Chinese Medicine, Beijing, China; 4 Beijing University of Chinese Medicine Collaborating Center of Joanna Briggs Institute, Beijing, China; 5 Beijing University of Chinese Medicine Best Practice Spotlight Organization, Beijing, China; 6 School of Epidemiology and Public Health, University of Ottawa, Ottawa, Ontario Canada; 7 Department of Nurse Anesthesia, Virginia Commonwealth University, Richmond, Virginia USA; 8 Lawrence S. Bloomberg Faculty of Nursing, University of Toronto, Toronto, Ontario Canada

**Keywords:** Knowledge translation, Implementation science, Theory, Model, Framework, China, Scoping review, Integrated knowledge translation

## Abstract

**Background:**

Knowledge translation (KT) theories, frameworks, and models (TFMs) can help guide and explain KT processes, and facilitate the evaluation of implementation outcomes. They play a critical role in conducting KT research and practice. Currently, little is known about the usage of TFMs in KT in Chinese healthcare settings. The aim of this scoping review is to identify which TFMs had been used for KT in healthcare settings in China, and how these TFMs were used.

**Methods:**

The protocol for this scoping review is in accordance with the Arksey and O’Malley framework and further enhanced by the recommendations suggested by Levac et al. We will search 8 databases (4 Chinese and 4 English) to identify relevant studies. Four reviewers (2 for Chinese, 2 for English) will independently screen studies based on the eligibility criteria. The basic characteristic of studies and the TFMs utilization (i.e., what, why, and how) will be extracted. Methodological quality and reporting quality will be assessed using the Mixed Method Appraisal Tool (MMAT) and the Standards for Reporting Implementation Studies (StaRI) (or Standards for Quality Improvement Reporting Excellence, SQUIRE 2.0 for quality improvement studies), respectively. All the retrieved TFMs will be categorized into Nilsen’s classifications of TFMs for KT research. We will employ the qualitative content analysis approach to summarize how these TFMs have been used, and the rationale. A consultation will be conducted through a 1-h interactive virtual meeting with an expert panel of knowledge users.

**Discussion:**

By conducting this scoping review, we expect to gain a comprehensive and in-depth understanding of why and how TFMs have been used in KT research and practice in China, and to identify gaps and provide recommendations for more efficient and meaningful use of TFMs in the future.

**Systematic review registration:**

This review has been registered with the Open Science Framework (10.17605/OSF.IO/8NXAM).

**Supplementary Information:**

The online version contains supplementary material available at 10.1186/s13643-020-01567-4.

## Background

Globally, the uptake of high-value clinical procedures, technologies, and research findings has been slow and challenging [1, 2]. Reports have shown that it takes an average of 17 years for an organization to incorporate evidence-based practice (EBP) into routine health care practice [3–5]. Significant research to practice gaps exists in healthcare discipline, and many of which require immediate attention. Over the past two decades, researchers have been focusing on reducing the research to practice gap using knowledge translation (KT), which is defined as “a dynamic and iterative process that includes synthesis, dissemination, exchange and ethically sound application of knowledge to improve the health, provide more effective health services and products and strengthen the health care system” [6]. KT has been used interchangeably with other terms in the literature, such as implementation science, dissemination, and implementation. The increased attention and interest in using KT to reduce the knowledge to practice gap resulted in a surge of international organizations, global policies, and funding opportunities that focus on advancing KT research and advocating the use of KT strategies in practice [7]. For instance, *KT Canada* was launched to connect Canadian KT experts and bridge the severe gaps between healthcare research and practice. The national funding agency Canadian Institutes of Health Research (CIHR) also announced that all grant applications to CIHR should include a plan on KT in their proposals [8]. In the USA, organizations such as the National Implementation Research Network and the Society for Implementation Research Collaboration have been established to facilitate communication and collaboration between implementation research teams, researchers, and community providers to advance the science and practice of KT. In 2010, the National Institutes of Health (NIH) established a standing review committee for dissemination and implementation research in health, linking KT research at NIH to the strategic plan of the United States Department of Health and Human Services [9]. KT research has also gained rapid advancement with the development of TFMs (e.g., *Knowledge-to-Action Framework* [1]), implementation toolkits (e.g., *Toolkit: Implementation of Best Practice Guidelines* [10]), new research designs (e.g., stepped wedge cluster randomized trials [11], effectiveness-implementation hybrid designs [12]), and reporting standards (e.g., *Standards for Reporting Implementation Studies (StaRI)* [13–15]).

The surge in national collaborative initiatives in North America greatly contrasts with the limited number of organizations that are dedicated to KT research in China. A few of the affiliation centers of Joanna Briggs Institute (JBI) (e.g., Fudan University JBI center, Peking University JBI center, Beijing University of Chinese Medicine JBI center) have begun to promote the awareness of KT research. Further, several hospitals and universities in China are enlisted as Registered Nurses’ Association of Ontario’s Best Practice Spotlight Organizations, with the primary goal to implement nursing best practice guidelines in clinical settings and academic institutes [16]. Lanzhou University established the World Health Organization Collaborating Centre for Guideline Implementation and Knowledge Translation. Despite recent advancement in the promotion and acceptance of KT research in China, the scientific research and practice on KT are still in its infancy. A scoping review synthesizing China’s implementation literature in nursing identified 96 papers [17]. Eighteen were found to have adopted a TFM to guide the study. However, no detailed information was obtained on why and how these TFMs were used [17]. To our knowledge, the medical literature on KT research and practice in China has not been synthesized. One previous study examining the published contents of the Chinese Journal of Evidence-Based Medicine (EBM) identified only two KT research papers, both addressing guideline implementation [7].

The importance of using TFMs in KT studies cannot be overstated. Theories in the KT context are often those explaining the causal mechanisms of implementation with predictive capacity on specific health behavior. Models are those describe the KT process. Frameworks are the factors influencing the implementation outcomes [18]. While it should be noted that it is not always distinguishable between theories, models and frameworks, and KT researchers may use them interchangeably. TFMs play a significant role in understanding the success and failure of implementation initiatives. They can help uncover the causal determinants of behavior change and make interventions more likely to be effective. Through the evaluation of theory-informed interventions, we can test and develop current theories and make it more adaptable across different context, population, and behaviors [19]. Nilsen grouped the KT TFMs into five categories consisting of three distinct goals [18]. Process models (e.g., *Knowledge-to-Action Framework* *[*1*]*) describe and/or guide the process of translating research into practice; determinant frameworks (e.g., *Theoretical Domain Framework* *[*20*]*), classic theories (e.g., *Theory of Diffusion* *[*21*]*), and implementation theories (e.g., *Normalization Process Theory* *[*22*]*) help understand and/or explain the determinates influencing implementation outcomes; evaluation frameworks can be used for implementation evaluation (e.g., *RE-AIM Framework* *[*23*]*). StaRI has clearly stated that it is necessary to report the “scientific background and rationale for the implementation strategy, including any underpinning theory, framework, or model, how it is expected to achieve its effects, and any pilot work” [14]. Hull et al. developed the Implementation Science Research Development tool, in which the use of implementation TFMs was listed as one of the ten indispensable domains [24]. In a scoping review of TFMs used in KT, 159 TFMs were found to inform the design, implementation, or evaluation of KT [25]. In Tabak et al.’s review, a total of 61 models were retrieved to assist researchers in enhancing their KT research [26]. Colquhoun et al. conducted a scoping review to examine the application of three KT theories: *Diffusion of Innovations*, *Promoting Action on Research Implementation in Health Services Framework*, and *Theory of Planned Behavior*. They found that these three theories were applied in KT research papers in five different ways: (1) a general philosophical framework, (2) a guide to educational KT strategies, (3) to identify variables for correlation or prediction, (4) to identify variables to measure the effect of a KT strategy, and (5) a guide to qualitative study design or analysis [27].

Despite a plethora of KT TFMs, few empirical studies adopt a theory-based approach for intervention development [25], which not only prevents further theory development but may also lead to failed implementation [28, 29]. Strifler and colleagues found that of the 159 TFMs, most of them were used in less than 1% of the included studies and many of them have been used only once [25]. It is highly suggested that rather than developing new TFMs, more efforts are required to test, refine, and integrate theories concretely with research and practice [29].

In China, KT gained much more popularity in nursing than other healthcare disciplines; however, a dearth of KT-based theoretical publications exist [17]. Zhou et al. [30] integrated the plan-do-study-action quality improvement tool with KT and generated a *Framework of Evidence-based Continuous Quality Improvement* to inform the clinical qualitative improvement initiatives. Ge et al. [31] conceptualized a *Researcher-Manager-Practitioner Collaborative Working Model of Evidence-Based Practice*, which described the roles and functions of researchers, managers, and practitioners in promoting the evidence application in real-world practice [32]. In addition, Hu et al. [33] and Yan et al. [34] have also developed the pathways on evidence-based nursing practice and guideline implementation, respectively. Since all the above TFMs are developed in the recent 5 years, they have not been extensively used in empirical studies. Further, to our knowledge, no reviews were completed to examine their usage. Due to language barriers, Chinese KT studies were seldom included in reviews conducted by foreign scholars. Strifler et al. retrieved 31 studies in Asia using TFMs in their KT research, but these were all English literature and no information was available on how many of these papers were from China [25]. Cheng’s [17] scoping review identified that within the 93 nursing KT studies, only 18 papers (19.35%) used theoretical frameworks, within which 13 adopted the *JBI Model of Evidence-Based Healthcare* *[*35*]*, two applied *Ottawa Model for Research Use* *[*36*]*, two used *ACE Star Model of Knowledge Transformation* *[*37*]*, and one used the *Knowledge-to-Action Framework* *[*1*]*. All these TFMs are process models [18] that guide the KT process. No other TFMs were used to examine the determinants or causal mechanism of implementation or evaluate the outcome. Further, little information was available from the review on the rationale of using a certain TFM and how it was used. Henceforth, a review to understand how KT TFMs have been used in the context of healthcare in China is warranted to (1) provide a more accurate representation of the extent to which KT TFMs were used and (2) explain how and why these KT TFMs were used.

## Aim

The purpose of the scoping review is to examine the evidence on the utilization of KT TFMs in the Chinese healthcare context. Specifically, we aim the following:

1. Identify published literature on the extent to which KT TFMs were applied;
2. Describe and explain the reasons why the KT TFMs were applied;
3. Appraise the methodological quality and reporting quality of the included literature.

## Methods

### Study design

The scoping review will be guided by the Arksey and O’Malley framework [38] and the recommendations by Levac et al. [39]. The PRISMA-ScR will be followed when reporting the review [40]. We choose the scoping review method because (1) scoping review helps to identify the types of available evidence in a given field [41]: No published literature is available on the status of KT TFM usage in Chinese healthcare settings; (2) scoping review examines how research is conducted on a certain topic or field [41]: We include literature with all study designs in our research to understand what, why, and how certain TFMs were used; (3) scoping review identifies and analyses knowledge gaps [41]: By conducting this scoping review, we aim to gain comprehensive and in-depth understanding on how TFMs were used in Chinese KT research so that usage gaps can be identified and recommendations be provided correspondingly. This review has been registered with the Open Science Framework (osf.io/7by5x).

The six steps of the scoping review will be followed:

### Identifying the research questions

As discussed earlier, this scoping review will examine the use of KT TFMs in the Chinese healthcare context. Specifically, we will identify literature on the extent to which KT TFMs were applied, the rationale of the TFMs application, and the methodological and reporting quality of included literature. We will consider KT from two different perspectives [25]: implementation practice (i.e., implementing the research evidence into practice) and implementation research (defined as the “scientific study of methods to promote the systematic uptake of research findings and other evidence-based practices into routine practice” [42]).

### Identifying relevant studies

Four Chinese databases, China National Knowledge Infrastructure, Wan Fang Database, The VIP Database, and China Biology Medicine and four English databases: Ovid MEDLINE, the Cumulative Index to Nursing and Allied Health Literature (CINAHL), EMBASE, and JBI Evidence-based Practice Database will be searched from 1996 (this year was chosen as the starting year due to the prevalence of KT publications since that time [27, 43]) to present. We will not search for gray literature in our review since KT research is still in its infancy in China, and we do not expect many unpublished reports, documents, or papers available. The search strategy is developed and determined by consulting one Chinese and one English librarian, respectively, combining with the input of the research team. See Additional file 1 for the initial search strategy of English literature from Ovid MEDLINE. A PRISMA diagram will be completed to record the number of articles identified, screened, and included for full-text review [44].

Publications must meet all of the following inclusion criteria:

- Studies focused on KT research or practice related to healthcare professionals in primary or acute care, clinical, or public health settings in China;
- Studies aimed to improve the dissemination, implementation, sustainability, or scaling up of evidence-based healthcare interventions using at least one TFM;
- Any study design with original data;

We will exclude publications that:

- Conducted KT research in educational institutes, such as medical colleges;
- Mentioned a KT TFM (e.g., in the introduction or discussion part) without using it to guide the implementation;
- Described the development or discuss the use of a KT TFM;
- Were book chapters, conference abstracts, reviews, commentaries, or study protocols;

### Study selection

All the retrieved publications from the above 8 databases will be imported into Covidence (https://www.covidence.org/), a web-based platform that streamlines the production of different types of literature reviews with the function of importing, screening, and exporting literature, to eliminate duplications and conduct literature screening. Four review authors (XL and LY for English papers, YY and WC for Chinese papers) will independently evaluate study eligibility in a two-stage screening process. First, titles/abstracts will be randomly assigned to two reviewers, and the reviewers will decide the relevance of these papers with three options “Yes”, “No,” or “Maybe.” Articles will be screened out when rated as “No” by both reviewers. If the title/abstract is considered “Yes” or “Maybe” by at least one reviewer, the article will enter the second screening stage. In the second stage, full texts of these potentially eligible studies will be retrieved and independently assessed for eligibility by two reviewers in duplicate. Disagreements will be discussed and resolved by consensus or with a third member (JZ) of the research team when necessary.

### Charting the data

A preliminary data extraction form was set up by the research team, and a pilot test of the form had been conducted with 5 Chinese articles and 2 English articles. A standardized data extraction form was developed after revision. Four review authors (XL and LY for English papers, YY and WC for Chinese papers) will independently extract the data with this form. Each review author will do half of the included papers and cross-check. Information will be extracted based on the Cochrane checklist for data extraction. Information extraction includes (1) study identification: publication year, authors, journal, language, and funding support; (2) methods: study design, study setting, and study duration; (3) participants: specialty and sample size; (4) implementation: implementation determinants and implementation strategies; (5) outcomes: patient outcomes, healthcare professional outcomes, and organizational outcomes; and (6) the use of TFMs: what TFM was used, why was it used, how was it used, and comments on its use (optional).

It is still under high debate on whether to include quality assessment into scoping review [39, 41, 45, 46]. In our study, after discussion with team members, we recognized that it was necessary to conduct the quality appraisal on those included papers for the fact that currently there was no published paper describing the quality of KT studies in China. It is valuable to gain understanding of the methodological quality and reporting quality of those published papers to inform future KT design and reporting. For each included paper, the methodological quality will be independently assessed by two reviewers (JZ, JH for English papers and WC, YY for Chinese papers) using the MMAT. MMAT can be used to concurrently appraise qualitative, quantitative, and mixed-method studies for large and complex reviews [47, 48]. All the reviewers have received formal training on critical appraisal. To improve the consistency between reviewers, a pilot quality appraisal will be conducted with ten included papers. Each reviewer will independently assess the quality and discuss the discrepancies afterwards. During the formal assessment, disagreement on the quality appraisal results will be resolved by discussion or by a third reviewer (SL). Reviewers will also assess the reporting quality of those included studies with StaRI [14, 15]. Some authors classified KT practice in their studies as organizational quality improvement initiatives. For those studies, SQUIRE 2.0 will be used as an appraisal tool instead of StaRI [49]. The reporting quality assessment will be conducted by one reviewer ((JZ for English papers and WC for Chinese papers) and checked by the second reviewer (JH for English papers and YY for Chinese papers).

### Collating, summarizing, and reporting the results

First, the number of included studies and basic characteristics of those included studies such as study designs, settings, and specialties will be reported. We will refer to Nilsen’s distinctions of TFMs in KT [18] and group TFMs into five categories: (1) process models, (2) determinants frameworks, (3), classic theories, (4) implementation theories, and (5) evaluation frameworks. The frequency of each TFM used will be counted. We will analyze the TFMs from three different perspectives: (1) why the TFM was chosen (the rationale), (2) how was it used, and (3) comments on its usage (e.g., the strength and/or limitations of the reporting and actual use of TFM in the paper). As suggested by Levac and colleagues [39], synthesizing process information, such as the use of a TFM, may benefit from the use of a qualitative content analysis approach to make sense of the wealth of extracted data. We will synthesize extracted data descriptively on the above information follow the four-step content analysis approach: (1) read all data repeatedly to achieve immersion and obtain a sense of the whole, (2) highlight the exact words from the text that appear to capture key thoughts or concepts, (3) build the initial code schemes, and (4) develop descriptive categories [50]. All the processes will be conducted manually in the Microsoft Excel form.

### Consultation

Even though Arksey and O’Malley suggest the consultation stage to be optional, it is recommended by Levac et al. that it is a required component to improve the methodological rigor [39]. From an integrated KT lens, involving knowledge users in the research process is also critical to generate more relevant and applicable knowledge and a greater capacity for and the likelihood of implementation [51]. In fact, some of the authors in our team have dual roles as both KT researchers (i.e., undertaking scientific research in KT field) and knowledge users (who have conducted or are conducting KT practice in clinical settings). The dual roles cannot only guarantee the rigor of the review from a scientific research perspective but also help shape the research process and findings to become more relevant. As to this consultation phase, we will engage additional knowledge users with the establishment of an expert panel consisting of KT researchers in our team and healthcare professionals (clinical nurses, physicians, and nurse managers) who have conducted or are going to conduct KT practice in their healthcare settings. The convenient sampling approach will be used by contacting the research team’s clinical partners and inquiring about their interest in joining this panel. To facilitate a meaningful and engaged conversation among panel members, we will only recruit five healthcare professionals. We will share our preliminary research findings with the panel in advance and hold a 1-h interactive virtual meeting with them. The aim of this meeting will be to (1) make sure that our findings are clear, understandable, and relevant to them all; (2) validate the findings and identify the research gaps; and (3) generate a list of key recommendations on the use of TFMs in KT and the research priorities in the future in this area.

## Discussion

This will be the first scoping review to examine the literature reporting the use of TFMs in KT in Chinese healthcare settings. Through this review, we can gain a comprehensive and in-depth understanding of why and how KT TFMs have been used, identify existing gaps, and provide recommendations for more efficient and meaningful use in the future. Moreover, we will adopt an integrated KT approach for our review. Our research team members have dual roles as both KT researchers and knowledge users. The interaction and contributions of team members, as outlined in this protocol, will ensure a robust and rigorous review process. We will also establish an expert panel with additional knowledge users in the consultation stage to make sure the results are understandable and usable. However, there are some potential limitations in the proposed review. First, due to the multifarious terms used to describe KT, we may miss relevant publications. Further, we will not be considering gray literature in our review based on the reasons outlined in our protocol. The output of this review will be a list of TFMs used in KT research and practice in China, a description of the methodological quality and reporting quality of those studies, and relevant recommendations to inform KT agendas in China. The research findings will be disseminated in a scientific journal and presented at academic conferences. Moreover, we will develop an e-version leaflet to provide key messages based on our review findings and disseminate it via social media. The institution that two team members (XL and LY) are affiliated has the official website (http://ebn.bucm.edu.cn/) providing KT resources for Chinese KT researchers and practitioners, we will apply to upload the research papers and the e-leaflet on this website.

## Supplementary Information

**Additional file 1.** Search strategy in Ovid MEDLINE.

## Data Availability

Not applicable

## Abbreviations

CIHR: Canadian Institutes of Health Research
CINAHL: Cumulative Index to Nursing and Allied Health Literature
EBM: Evidence-based medicine
EBP: Evidence-based practice
JBI: Joanna Briggs Institute
KT: Knowledge translation
MMAT: Mixed Method Appraisal Tool
NIH: National Institutes of Health
PRISMA-ScR: Preferred Reporting Items for Systematic Reviews and Meta-Analyses extension for scoping reviews
SQUIRE: Standards for Quality Improvement Reporting Excellence
StaRI: Standards for Reporting Implementation Studies
TFMs: Theories/frameworks/models
