# Supplementary material for: The use of theories, frameworks, or models in knowledge translation studies in healthcare settings in China: a scoping review protocol
Source: Syst Rev. 2021 Jan 7;10:13. doi: 10.1186/s13643-020-01567-4 (PMC7792291; doi:10.1186/s13643-020-01567-4)
Supplement: Supplementary file 1 — Additional file 1. Search strategy in Ovid MEDLINE. [file 13643_2020_1567_MOESM1_ESM.docx]

**Search strategy--- Ovid MEDLINE**

Search for: limit 22 to yr="1996 -Current"
Results: 465

Database: Ovid MEDLINE(R) ALL <1946 to August 16, 2019>
Search Strategy:
--------------------------------------------------------------------------------
1     exp Translational Medical Research/ (9707)
2     exp Evidence-Based Practice/ (84999)
3     exp organizational innovation/ (25894)
4     exp guideline adherence/ (30325)
5     exp implementation science/ (168)
6     exp diffusion of innovation/ (19113)
7     Knowledge [translation.mp](http://translation.mp/). (2746)
8     knowledge [transfer.mp](http://transfer.mp/). (1682)
9     knowledge utili*[ation.mp](http://ation.mp/). (120)
10     knowledge mobili*[ation.mp](http://ation.mp/). (115)
11     knowledge [exchange.mp](http://exchange.mp/). (557)
12     ((evidence based or evidence informed) adj3 (practice or nursing or medicine or healthcare or health care)).mp. (98946)
13     [dissemination.mp](http://dissemination.mp/). (70331)
14     (organi*ation* adj3 innovat*).mp. (24448)
15     (guideline adj3 (implementation or utili*ation)).mp. (1921)
16     (implementation adj3 (science* or research)).mp. (5882)
17     (diffusion adj3 innovation).mp. (17508)
18     (research adj3 utili*ation).mp. (1684)
19     1 or 2 or 3 or 4 or 5 or 6 or 7 or 8 or 9 or 10 or 11 or 12 or 13 or 14 or 15 or 16 or 17 or 18 (251541)
20     (theory or model or framework or cycle).mp. (2872651)
21     (China or Chinese).mp. (389649)
22     19 and 20 and 21 (474)
23     limit 22 to yr="1996 -Current" (465)
